# Student satisfaction in transnational higher education in China: A mixed-methods study across Australian, German, and US cooperative programmes

**DOI:** 10.1371/journal.pone.0347655

**Published:** 2026-04-21

**Authors:** Mingzheng Hu

**Affiliations:** Moray House School of Education and Sport, University of Edinburgh, Edinburgh, United Kingdom; Bangor University, UNITED KINGDOM OF GREAT BRITAIN AND NORTHERN IRELAND

## Abstract

This study examines student satisfaction in Sino-foreign cooperative programmes at a Chinese university with partner institutions from Australia, Germany, and the United States. Using a mixed-methods design that combines survey data from 121 undergraduates with 10 semi-structured interviews, it explores whether satisfaction varies across student backgrounds and programme characteristics, and identifies the factors most closely associated with these evaluations. Most participants (87.6%) were enrolled in the 4 + 0 non-mobile pathway, indicating that non-mobile participation constituted the dominant form of transnational higher education (TNE) engagement in this institutional context. Quantitative analysis shows no significant variation by gender, year, or place of origin, but significant differences by major, parental education, family income, and foreign partner institution. Correlation and interview findings suggest that satisfaction was most strongly associated with the teaching system, learning attitudes, and the quality of teacher-student and peer relationships, while motivation showed a more moderate association. The study contributes theoretically by showing that student satisfaction in Chinese TNE is best understood as a multidimensional and socially situated evaluation, shaped by programme design, family resources, and everyday educational experience rather than by international branding alone.

## 1. Introduction

Transnational higher education (TNE) has become a central feature of global higher education in the twenty-first century. As Altbach [1] and Knight [2] argue, TNE is both a product of and a response to globalisation, reshaping the flow of knowledge, talent, and educational services across borders. It takes diverse forms, from branch campuses and franchised programmes to dual-degree and joint-institute models. While these initiatives have expanded access and built institutional capacity, they also raise critical debates around dependency, inequality, and sustainability [3–6]. Thus, TNE has attracted both advocates, who view it as a vehicle for knowledge transfer and global engagement, and critics, who question its uneven benefits and local impacts.

China has emerged as the world’s largest host of TNE, reflecting both state policy and market demand. Since the 2003 Regulations on Sino-foreign Cooperative Education, the number of cooperative programmes and institutions has exceeded 2,300 [7]. These programmes are framed as a strategic means to internationalise Chinese higher education, introduce advanced foreign curricula, and enhance global competitiveness [8]. Unlike mobility-based models, many of these initiatives provide non-mobile pathways such as 4 + 0, enabling students to complete their entire studies in China while receiving dual or foreign degrees. This form of local internationalisation has proven especially attractive to students and families concerned with the costs, risks, and cultural barriers of overseas study [9].

Despite this rapid expansion, research on Sino-foreign cooperation has largely prioritised governance, policy frameworks, and institutional arrangements [10–13]. Although some recent studies have examined student identity, aspirations, and intercultural development, less attention has been paid to how undergraduates evaluate the quality of their programme experience while still enrolled. This question is especially important in non-mobile pathways, where students engage with international curricula locally rather than through overseas study.

The literature on student satisfaction in TNE remains relatively limited. Existing studies suggest that satisfaction is shaped not only by teaching quality and institutional provision but also by whether students perceive their experiences as meeting prior expectations [14,15]. However, much of this work relies on large-scale quantitative surveys [16], which are useful for description but less effective in capturing how students interpret, justify, or question their experiences in context. As a result, student satisfaction is often measured more clearly than it is explained.

Although TNE has expanded rapidly, three related gaps remain in the literature. First, existing studies continue to focus disproportionately on student mobility, international branch campuses, or postgraduate outcomes, while undergraduate experiences in non-mobile 4 + 0 pathways remain comparatively underexplored [17]. This reflects a broader tendency to equate internationalisation with cross-border movement, overlooking the growing significance of locally delivered international programmes in host contexts such as China.

Second, research on student satisfaction in TNE is still dominated by quantitative survey designs that treat students primarily as respondents to pre-set indicators. While such studies provide useful descriptive patterns, they often say less about how students interpret their experiences, reconcile expectations with actual programme conditions, or make sense of dissatisfaction. Mixed-methods approaches that combine patterned comparison with student voice remain relatively limited.

Third, comparative analysis within the same host institution is rare. Prior studies often focus on a single partnership or programme, making it difficult to distinguish what is specific to one foreign collaboration from what is shaped by the shared local context. Yet in China, it is increasingly common for one university to host multiple cooperative programmes with different foreign partners. This makes the Chinese context especially valuable for examining how curriculum, pedagogy, reputation, and organisational arrangements interact within a common institutional environment.

This study addresses these gaps by examining undergraduate satisfaction in one Chinese university that operates cooperative programmes with partners from Australia, Germany, and the United States. The study treats satisfaction as a multidimensional evaluative perception of students’ programme experience rather than as a simple proxy for objective quality. Using a mixed-methods design that combines questionnaires with semi-structured interviews, it identifies variation across student backgrounds and programme characteristics while also examining how students themselves explain these evaluations.

Although multiple background and programme variables are considered, the analysis remains focused on characteristics most closely linked to the conceptual framework, particularly family resources, programme environment, and partner-related institutional differences. Accordingly, the study asks two questions:

1 Are there differences in undergraduate student satisfaction across student backgrounds (e.g., gender, academic year, parental education, family income) and programme characteristics (e.g., major, foreign partner) within Sino-foreign cooperative programmes in one Chinese university?
2 What aspects of undergraduates’ experiences are most closely associated with student satisfaction in these cooperative programmes?

## 2. Theoretical Framework

No single framework is sufficient to explain student satisfaction in Sino-foreign cooperative programmes. Institutional models such as the Input-Environment-Outcome (I-E-O) framework help identify how student background and programme environments relate to outcomes, but they pay limited attention to students’ subjective evaluations. Expectancy-disconfirmation theory explains how students judge their experience against prior expectations, yet it is less attentive to structural inequality. Cultural capital highlights how family resources and stratification shape educational experience, but it cannot on its own explain variation within the same institutional setting. For this reason, this study integrates three complementary perspectives: Astin and Antonio’s [18] I-E-O model, Oliver’s [19] expectancy-disconfirmation theory, and Bourdieu’s [20] concept of cultural capital.

### 2.1 Input-Environment-Outcome (I-E-O) model

Astin & Antonio’s [18] I-E-O model emphasises how student characteristics (input), institutional and social experiences (environment), and learning results (outcome) interact. Inputs include demographic variables such as gender, parental education, and family income, while the environment covers teaching practices, peer networks, and institutional systems. Outcomes in this study refer to students’ perceived satisfaction. The model underscores the mediating role of the environment: outcomes are not predetermined by inputs but shaped by how students experience their institutional setting [21]. However, while the I-E-O model maps structural relationships well, it says little about how students subjectively evaluate their experiences, making it insufficient as a stand-alone framework.

### 2.2 Expectancy-disconfirmation theory

Expectancy-disconfirmation theory [19] explains satisfaction as a psychological state derived from comparing expectations with perceived performance. When performance exceeds expectations, satisfaction rises; when it falls short, dissatisfaction follows. Adapted to higher education [14], the theory helps explain why students within the same environment may diverge in their evaluations, depending on whether the programme meets or frustrates their expectations. In TNE contexts, where families often invest heavily with hopes of “international quality,” expectation-experience alignment is particularly salient. Yet this framework remains narrowly individualistic, neglecting how expectations themselves are socially constructed. It therefore requires supplementation by a sociological perspective.

### 2.3 Cultural capital

Bourdieu’s [20] concept of cultural capital situates educational experiences within broader patterns of inequality and reproduction. Parents’ education levels and broader family educational resources shape students’ aspirations, learning dispositions, confidence, and ability to mobilise institutional opportunities. In Chinese higher education, family cultural capital has been linked to differences in educational engagement and outcomes, making it a useful lens for understanding why students may evaluate the same programme differently. In this study, cultural capital is used to interpret how family background may shape both students’ expectations of TNE and the criteria through which they judge programme quality.

### 2.4 Integrating the three perspectives

By combining these frameworks, the study avoids the limitations of any single approach. The I-E-O model explains how inputs and environments interact to shape outcomes, expectancy-disconfirmation theory highlights the psychological mechanisms of expectation alignment, and cultural capital situates those expectations and evaluations within broader family and social contexts. Together, they provide a robust framework for understanding how undergraduate satisfaction in cooperative programmes is shaped at the intersection of background, institutional design, and subjective interpretation.

## 3. Methodology

This study employed a mixed-methods case study design to investigate undergraduate satisfaction in Sino-foreign cooperative programmes at one Chinese university. A mixed-methods approach was considered appropriate because it combines the breadth of quantitative surveys with the depth of qualitative inquiry, allowing for a more comprehensive understanding of student satisfaction [22]. In particular, while large-scale survey data enabled the identification of general patterns and group level differences, the interviews offered insights into students’ expectations, lived experiences, and interpretations [23]. This design responds to critiques of TNE satisfaction research that highlight its over reliance on quantitative indices while neglecting the voices of students themselves [15].

The case university provides an especially suitable setting for this study, as it hosts multiple Sino-foreign cooperative programmes in collaboration with universities from Australia, Germany, and the United States. Although these partnerships vary in terms of curriculum design, teaching resources, and reputational capital, they share the distinctive feature of offering 4 + 0 non-mobile pathways, enabling students to complete their degree entirely in China while receiving dual degrees. Optional mobile pathways (2 + 2 and 3 + 1) are also available. This institutional configuration offers a unique opportunity to examine how student satisfaction is shaped across diverse cooperative programmes in the same Chinese host institution context. The case was bounded as one International Business School within a Chinese university, comprising three undergraduate Sino-foreign cooperative programmes with partner institutions from Australia, Germany, and the United States. The case therefore captures variation across foreign partners and disciplinary arrangements within a shared institutional and local context, while excluding non-cooperative programmes elsewhere in the university.

Participants were recruited between November 2023 and February 2024 through undergraduate transnational education programmes at a university in China. The quantitative component consisted of a survey administered to undergraduates enrolled in the International Business School, which oversees all three cooperative programmes. Freshmen were excluded, as their limited time in the programme was unlikely to provide sufficient experience to form reliable evaluations. In total, 123 questionnaires were collected, of which 121 were valid, producing a response rate of 98.4%. The high response rate reflects the use of programme-level administrative assistance and established student communication channels during distribution. Participation was voluntary and anonymous, and completion of the survey had no connection to course assessment or formal programme evaluation. At the same time, institutionally mediated distribution may have increased participation among students who were more engaged with programme communication, and this possible response bias should be acknowledged.

Student satisfaction was operationalised as a multidimensional evaluative perception of programme experience. Questionnaire items were adapted from established higher education student satisfaction research, particularly studies that conceptualise satisfaction in relation to teaching and learning, facilities, support, and perceived benefits [14,16]. In this study, the four dimensions were classroom activities, extracurricular activities, campus facilities, and perceived benefits. These dimensions were selected to reflect both academic and non-academic aspects of student experience in Sino-foreign cooperative programmes. All satisfaction items were measured on a five-point Likert scale ranging from “very dissatisfied” to “very satisfied”. Reliability tests indicated a high level of internal consistency (Cronbach’s α = 0.895), with all sub-dimensions exceeding the acceptable threshold of 0.60 [24]. Survey data were analysed using SPSS to generate descriptive statistics and to conduct inferential tests, including ANOVA and chi-square to identify significant differences by student background and programme characteristics [25]. Pearson correlation coefficients were used to examine the relationships between satisfaction and potential influencing factors [26].

The qualitative component consisted of ten semi-structured interviews, conducted online via Zoom or WeChat and lasting between 30 and 60 minutes. A purposive sampling strategy was used to ensure variation across gender, socio-economic background and majors. Students were given the option to be interviewed in either English or Mandarin, with all interviews recorded, transcribed, and anonymised. Table 1 provides the profile of the 10 interview participants, highlighting variation in gender, academic year, major, and pathway. This diversity reflects the structural differences across the cooperative programmes, which is central to examining satisfaction patterns.

**Table 1:** Profile of semi-structured interview participants.

Pseudonym	Gender	Academic Year	Major	Programme
Jason	Male	Second	China-Australia Accounting	4 + 0
Xander	Male	Second	China-Australia International Economics and Trade	4 + 0
Helena	Female	Third	China-US Computer Science and Technology	4 + 0
Steven	Male	Fourth	China-Australia International Economics and Trade	4 + 0
Winter	Female	Second	Sino-German International Economics and Trade	4 + 0
Gemma	Female	Third	China-Australia Accounting	2 + 2
Liberty	Female	Third	China-US Measurement and Control Technology and Instruments	4 + 0
Abby	Female	Fourth	China-US Business Administration	4 + 0
Andy	Male	Fourth	Sino-German Biological Engineering	4 + 0
Cambell	Female	Fourth	China-Australia Computer Science and Technology	2 + 2

The interview guide included questions on students’ expectations prior to entering the programme, their experiences of teaching, learning, and campus life, and their overall evaluations of satisfaction. Interview data were analysed using thematic analysis [27]. Initial coding was informed by the conceptual framework, particularly the distinction between student background, programme environment, and evaluative outcomes, while allowing for inductive coding where participants raised issues not fully captured by the pre-existing categories. All interviews were coded by the author. For Mandarin interviews, transcription and translation were also conducted by the author, with repeated checking against the original recordings to preserve meaning. To enhance analytical transparency, a coding log and theme-development notes were maintained throughout the analysis, and reflexive memoing was used to record key interpretive decisions.

Ethical approval for the study was obtained in accordance with institutional guidelines. All participants provided informed consent. Written informed consent was obtained from interview participants. For the questionnaire, participants were presented with an information sheet and consent statement before accessing the survey, and completion of the questionnaire was taken as informed consent. Participation was voluntary and anonymous, and all data were anonymised prior to analysis. Pseudonyms were used in reporting the qualitative findings, and all identifying information was removed to protect the anonymity of participants.

## 4. Findings

This section presents the results of the questionnaire (n = 121) and interviews (n = 10), structured around the two research questions. Survey results provide an overview of satisfaction levels and highlight differences across student backgrounds and programme characteristics (RQ1), while correlation analysis and interviews reveal the key factors shaping satisfaction (RQ2). Specifically, survey results reveal that 87.60% of participants chose the non-mobile 4 + 0 pathway, indicating that the majority of undergraduates in these cooperative programmes preferred to complete their studies entirely in China. This distribution underscores the importance of focusing on non-mobile pathways when analysing undergraduate satisfaction in Sino-foreign cooperative programmes.

### 4.1 Differences in student satisfaction across backgrounds and programme characteristics

Survey analysis shows that undergraduate satisfaction in the cooperative programmes was moderate across all dimensions. Table 2 indicates that campus facilities received the highest mean score (M = 2.98), followed by classroom activities (M = 2.77), perceived benefits (M = 2.76), and extracurricular activities (M = 2.68). These results suggest that students were broadly satisfied with teaching and physical resources, but less so with co-curricular opportunities.

**Table 2:** Overall state of student satisfaction (N = 121).

Variables	Curricular activities	Extracurricular activities	Campus facilities	Perceived benefits	Student experience
Mean value	2.77	2.68	2.98	2.76	2.81
The number of items	7	4	5	9	25

No significant differences were found by gender, academic year, or place of origin. Liberty, a Third-year student in the China-US Measurement and Control programme, confirmed this pattern: “We all go through the same courses and teachers, so our satisfaction is similar. The only difference is that seniors like me also have internships.” Andy, a fourth year in the Sino-German Biological Engineering programme, also noted: “Where students come from doesn’t matter much now, since education across regions is more balanced.” These perspectives indicate that demographic variables such as gender or origin exert limited influence.

However, significant differences appeared across majors, parental education, family income, and foreign partner programmes. As Table 3 shows, students in Computer Science (China-US) reported higher satisfaction with extracurricular opportunities than those in Business Administration (China-US). Helena, a Computer Science student, explained: “We had many competitions linked to our courses, which made extracurriculars meaningful and motivating.” By contrast, Abby, a fourth-year student in Business Administration, described: “Our workload was too heavy with endless assignments, so I didn’t have time for extracurriculars.” This demonstrates how disciplinary organisation and programme traditions shape opportunities for engagement.

**Table 3:** Differences in student satisfaction by major.

Dimensions	Major	N	Mean	F	Sig.
Curricular activities	China-Australia International Economics and Trade	24	2.1964	1.676	0.122
	China-Australia Accounting	21	2.2449		
	China-Australia Computer Science and Technology	20	2.1071		
	China-US Business Administration	10	2.0857		
	China-US Measurement and Control Technology and Instruments	14	2.4286		
	Sino-German International Economics and Trade	12	2.2143		
	Sino-German Biological Engineering	11	2.4805		
	China-US Computer Science and Technology	9	2.4603		
Extracurricular activities	China-Australia International Economics and Trade	24	2.6979	2.432	0.023
	China-Australia Accounting	21	2.7976		
	China-Australia Computer Science and Technology	20	2.3750		
	China-US Business Administration	10	2.1500		
	China-US Measurement and Control Technology and Instruments	14	2.8750		
	Sino-German International Economics and Trade	12	2.6458		
	Sino-German Biological Engineering	11	2.5682		
	China-US Computer Science and Technology	9	3.0833		
Campus facilities	China-Australia International Economics and Trade	24	2.6167	1.666	0.124
	China-Australia Accounting	21	2.5810		
	China-Australia Computer Science and Technology	20	2.4700		
	China-US Business Administration	10	2.5400		
	China-US Measurement and Control Technology and Instruments	14	2.8429		
	Sino-German International Economics and Trade	12	2.4833		
	Sino-German Biological Engineering	11	2.5273		
	China-US Computer Science and Technology	9	3.1778		
Perceived benefits	China-Australia International Economics and Trade	24	2.2824	1.692	0.118
	China-Australia Accounting	21	2.3545		
	China-Australia Computer Science and Technology	20	2.4167		
	China-US Business Administration	10	2.4333		
	China-US Measurement and Control Technology and Instruments	14	2.6667		
	Sino-German International Economics and Trade	12	2.4630		
	Sino-German Biological Engineering	11	2.5758		
	China-US Computer Science and Technology	9	2.8395		
Student experience	China-Australia International Economics and Trade	24	2.4340	1.196	0.311
	China-Australia Accounting	21	2.3770		
	China-Australia Computer Science and Technology	20	2.3813		
	China-US Business Administration	10	2.2583		
	China-US Measurement and Control Technology and Instruments	14	2.5565		
	Sino-German International Economics and Trade	12	2.3646		
	Sino-German Biological Engineering	11	2.5455		
	China-US Computer Science and Technology	9	2.6759		

Parental education, especially maternal education, was also significantly correlated with satisfaction (see Table 4). Students whose mothers had higher education reported greater satisfaction across curricular, extracurricular, and campus facility dimensions. Helena reflected: “My mother always encouraged me to take part in activities and make use of opportunities. That gave me more confidence and made me appreciate the programme.” By contrast, another interviewee from a less-educated family background noted: “My parents just told me to focus on classes. They never thought activities mattered.” These findings suggest that cultural capital is closely related to student orientations toward engagement.

**Table 4:** Differences in student satisfaction by parental education.

		Curricular activities	Extracurricular activities	Campus facilities	Perceived benefits	Student experience
Father’s Educational Level	F	1.661	2.118	2.354	2.298	2.978
	Sig.	0.137	0.056	0.035	0.039	0.010
Mother’s Educational Level	F	4.178	2.155	2.180	3.636	4.937
	Sig.	0.001	0.032	0.040	0.002	0.000

Family income produced a non-linear effect. As shown in Table 5, students from middle-income households (100,001–200,000 RMB annually) reported higher satisfaction than both lower and higher income peers. For lower-income students, tuition was burdensome: one explained, “It’s really expensive for my family, so sometimes I wondered whether it was worth it.” For higher-income students, dissatisfaction came from unmet elite aspirations. Andy commented: “I thought this programme would help me get into a top global university, but I could only apply to an average one. That gap lowered my satisfaction.” By contrast, Gemma, a 2 + 2 student in Accounting, highlighted value for money: “At first, I thought the fee was too high, but later I realised I gained more than I expected. For me, it was worth it”.

**Table 5:** Differences in student satisfaction by family income.

Dimensions	Family average year Income	N	Mean	F	Sig.
Curricular activities	Below ¥100,000	9	2.6667	4.840	0.010
	¥100001-¥200,000	66	2.2489		
	Above ¥200,000	46	2.1832		
Extracurricular activities	Below ¥100,000	9	3.0833	2.801	0.035
	¥100001-¥200,000	66	2.6742		
	Above ¥200,000	46	2.5272		
Campus facilities	Below ¥100,000	9	3.1111	3.981	0.021
	¥100001-¥200,000	66	2.6515		
	Above ¥200,000	46	2.4957		
Perceived benefits	Below ¥100,000	9	2.8642	7.169	0.001
	¥100001-¥200,000	66	2.5370		
	Above ¥200,000	46	2.2705		
Student experience	Below ¥100,000	9	2.7824	5.371	0.006
	¥100001-¥200,000	66	2.4634		
	Above ¥200,000	46	2.3297		

Differences also appeared across foreign partner programmes. Students in the China-US Computer Science programme rated extracurricular activities and facilities more highly than those in the China-Australia Business Administration programme. Helena noted the benefit of practice-oriented competitions in her US-affiliated programme, while Abby described how the heavy workload in her BA course limited engagement. These results show that programme design and partner traditions intersect with student agency to shape satisfaction, even within the same university.

In summary, RQ1 is answered by showing that satisfaction does not differ significantly by gender, year, or place of origin, but is strongly differentiated by major, parental education, family income, and foreign partner programme. These findings reflect how disciplinary organisation, cultural capital, and economic resources structure student opportunities and perceptions of value.

### 4.2 Influencing factors of student satisfaction

Correlation analysis revealed that satisfaction was strongly associated with the teaching system (r = .833), learning attitudes (r = .776), teacher-student relationships (r = .760), and peer relationships (r = .735), with motivation also positively correlated (r = .550). Table 6 summarises these results.

**Table 6:** Correlation analysis between satisfaction and influencing factors.

		Peer relationship	Teacher-student relationship	Motivation for learning	Learning attitude	Teaching system	Student satisfaction
Peer relationship	Pearson Correlation	1	.482^**^	.298^**^	.453^**^	.460^**^	.735^**^
	Sig. (2-tailed)		0.000	0.001	0.000	0.000	0.000
Teacher-student relationship	Pearson Correlation	.482^**^	1	.290^**^	.535^**^	.552^**^	.760^**^
	Sig. (2-tailed)	0.000		0.001	0.000	0.000	0.000
Motivation for learning	Pearson Correlation	.298^**^	.290^**^	1	.381^**^	.277^**^	.550^**^
	Sig. (2-tailed)	0.001	0.001		0.000	0.002	0.000
Learning attitude	Pearson Correlation	.453^**^	.535^**^	.381^**^	1	.699^**^	.776^**^
	Sig. (2-tailed)	0.000	0.000	0.000		0.000	0.000
Teaching system	Pearson Correlation	.460^**^	.552^**^	.277^**^	.699^**^	1	.833^**^
	Sig. (2-tailed)	0.000	0.000	0.002	0.000		0.000
Student satisfaction	Pearson Correlation	.735^**^	.760^**^	.550^**^	.776^**^	.833^**^	1
	Sig. (2-tailed)	0.000	0.000	0.000	0.000	0.000	

**. Correlation is significant at the 0.01 level (2-tailed).

The teaching system was repeatedly emphasised in interviews. Gemma, from the 2 + 2 Accounting programme, explained: “I have kept my GPA above 90 because it is necessary for postgraduate applications. That pushes me to make full use of the resources.” Jason, a Second-year student in Accounting, highlighted seminar participation: “We often divide into small groups to complete tasks, which improves teamwork and presentation skills. That makes learning more engaging.” These examples illustrate why the teaching system had the strongest correlation: it provided both incentives and interactive structures that were associated with higher satisfaction.

Learning attitudes also proved important. Cambell, who studied Computer Science (2 + 2), recalled: “I went to the library every night, and when I solved problems with classmates or teachers, I began to enjoy studying. That made me feel satisfied.” This shows how positive attitudes enable students to convert challenges into achievements.

Teacher-student relationships were highly valued. Liberty, in the Measurement and Control programme, reflected: “The foreign teachers were strict but fair. They encouraged us to think independently, which made me respect them and feel satisfied.” Yet some students reported frustrations with limited feedback from local staff. One remarked: “Sometimes teachers were too busy to respond, which made me less satisfied.” These contrasting experiences explain why teacher-student relationships were strongly, but not uniformly, correlated with satisfaction.

Peer relationships were another crucial factor. Steven, a fourth-year student of International Economics and Trade, described how friendships shaped his experience: “At first I couldn’t adjust, but later I became close to my roommates and even studied with my girlfriend. That made me more motivated and happier.” Winter, from the Sino-German programme, added: “Seeing seniors get offers from QS top universities motivated me to study harder.” These interview accounts suggest that peers act both as collaborators and as aspirational figures.

Finally, motivation also contributed, though more modestly. Xander, a Second-year student in International Economics and Trade, explained: “In my first year I joined many activities, but later I lost interest. That decline made me less satisfied.” This suggests that while motivation is important, it is fragile and may erode under workload or unmet expectations.

In summary, RQ2 is addressed by showing that student satisfaction was most strongly associated with the teaching system, learning attitudes, and the quality of teacher-student and peer relationships, while motivation showed a more moderate positive association. The interview data help interpret these relationships by showing how students experienced the everyday conditions through which satisfaction was formed and evaluated.

## 5. Discussion

This study examined undergraduate satisfaction in multiple Sino-foreign cooperative programmes within one Chinese university. The dominance of the 4 + 0 non-mobile pathway in this case indicates that the majority of undergraduates opted to complete their studies entirely in China. This highlights that non-mobile participation is not a marginal phenomenon but the dominant form of engagement in this institutional setting. The findings reveal that while demographic factors such as gender, academic year, and place of origin were not significantly associated with variation in satisfaction, satisfaction varied strongly by major, parental education, family income, and foreign partner. Furthermore, the factors most strongly associated with satisfaction were the teaching system, learning attitudes, and the quality of teacher-student and peer relationships. These findings resonate with, and also extend, existing scholarship on student experience in TNE.

### 5.1 Programme mechanics as the proximate drivers of satisfaction

In this discussion, “programme mechanics” refers to the everyday organisational features through which students experience the programme, including assessment arrangements, classroom interaction, feedback practices, academic support, and access to co-curricular opportunities. One of the most striking findings is that satisfaction was strongly associated with programme mechanics, particularly the teaching system (r = .833), and relational dynamics, including teacher-student and peer interactions. This supports the argument that satisfaction in TNE is related less to international branding alone and more to the organisation of pedagogy, assessment, and support. Haupt and Chelabi [15] and Rigopoulos [28], in their analysis of dual-degree programmes, emphasise that specific components such as assessment clarity, quality of feedback, and access to learning support services are the major correlates of student satisfaction, rather than the symbolic appeal of internationalisation. The findings suggest that student satisfaction in these Sino-foreign cooperative programmes was associated less with international branding alone than with the everyday mechanics of programme delivery. Teaching arrangements, classroom interaction, feedback practices, and peer and teacher relationships formed the most immediate conditions through which students evaluated programme quality. This helps explain why major and partner institution differences were more salient than gender or place of origin: students judged the programme primarily through experienced educational processes rather than through background characteristics alone.

### 5.2 Divergence across programmes within the same university

A second contribution of this study is the demonstration of significant divergence across majors and foreign partners within the same university. Students in the China-US Computer Science programme expressed higher satisfaction with extracurricular activities than those in the China-US Business Administration programme, attributing this to project-linked competitions in the former and heavy coursework in the latter. These findings support Cai et al.’s [29] argument that institutional mission and governance shape students’ identity and perceived value in TNE settings. While Cai et al. [29] compared international branch campuses with Sino-foreign institutes, this study highlights that even within one institution, partner traditions and disciplinary ecologies produce distinct satisfaction trajectories.

This observation also echoes Lai’s [8] analysis of “internationalisation with Chinese characteristics,” where global curricula are embedded in local governance and assessment systems, producing paradoxical student experiences. Students in this study valued English-medium seminars and peer role models, but some also reported anxiety stemming from workload intensity and linguistic challenges. By linking such paradoxes to measurable satisfaction outcomes, this study demonstrates how intra-institutional divergence, rooted in partner traditions and disciplinary demands, directly translates into student satisfaction differentials.

### 5.3 Socially conditioned expectations and the role of cultural capital

Beyond institutional factors, the results show that satisfaction is socially conditioned. Students with more highly educated mothers reported higher satisfaction across curricular, extracurricular, and campus dimensions. This aligns with Bourdieu’s [20] concept of cultural capital, which emphasises how family resources shape dispositions toward learning and the ability to mobilise opportunities [30]. The salience of maternal education has also been highlighted in Chinese higher education sociology, where it is consistently linked to students’ academic orientations and attainment [31]. The role of maternal education should not be interpreted deterministically. Rather, in this case it appears as a salient indicator of family cultural capital, helping to explain variation in students’ confidence, expectations, and evaluative frameworks. It is therefore better understood as one important pattern within a broader configuration of family resources, rather than as the sole source of variation in satisfaction.

Lai and Jung [17] note that students in Sino-foreign programmes often adopt a “master’s progression script,” expecting postgraduate study abroad as the natural next step. In this study, such expectations structured how students interpreted their undergraduate experience. High-income students voiced dissatisfaction when postgraduate outcomes fell short of elite aspirations, whereas middle-income students often described satisfaction when opportunities exceeded initial doubts. This reflects Oliver’s [19] expectancy-disconfirmation framework, but with a socially embedded twist: expectations are not neutral but mediated by family background and cultural capital.

The importance of intercultural communicative competence (ICC) as a mediating factor is also noteworthy. Xie [32] showed that ICC enhances Chinese students’ motivation in TNE partnerships. In this study, students who struggled with English-medium instruction described lower satisfaction, suggesting that ICC functions as a “conversion factor” [33], transforming exposure into confidence and confidence into satisfaction. Parental education may again play a role by scaffolding ICC development through early language support or cultural resources, thereby making institutional opportunities more legible and valuable to some students than others.

Read through a Bourdieusian lens, these patterns suggest that satisfaction in TNE is partly stratified by unequal access to cultural and economic resources. Such resources shape not only students’ capacity to participate in programme opportunities, but also the standards against which the programme is judged. This interpretation also resonates with wider TNE literature showing that internationalised provision can reproduce, rather than erase, educational inequality.

### 5.4 Implications

These findings have implications for how student satisfaction in TNE is conceptualised and evaluated. Rather than treating satisfaction as a simple reflection of institutional reputation or international branding, the study shows that it is closely tied to the everyday educational environment through which students encounter the programme. This suggests that evaluations of TNE quality should pay closer attention to teaching systems, relational support, and the actual conditions of student participation.

The study also contributes to ongoing debates about non-mobile internationalisation. In this case, the dominant form of TNE engagement was not overseas mobility but local participation in an internationalised programme. This indicates that non-mobile pathways should not be treated as secondary or residual forms of international education. Instead, they represent a central site through which questions of quality, inequality, and student experience in TNE need to be understood.

### 5.5 Limitations

While this study contributes new insights, some limitations should be noted. The analysis focused on one university, which allowed for detailed within-institutional comparison but limits generalisability. Future research across multiple universities and regions would clarify how institutional settings shape student satisfaction. The cross-sectional design provided clear patterns but did not capture how satisfaction evolves over time; longitudinal studies could follow students across their undergraduate years to trace changes in expectations and evaluations. Interviews enriched the analysis with student voices, but future work could also incorporate the perspectives of teachers, administrators, and families. Finally, although the programmes studied involved partners from Australia, the United States, and Germany, the analysis did not systematically compare national partner effects. Comparative studies across a broader range of partnerships would deepen understanding of how different academic traditions influence satisfaction. Nevertheless, by focusing on one university with multiple foreign partners, the study provides a unique within-institutional comparative lens rarely found in existing literature.

### 5.6 Future research

Future research would benefit from extending this analysis across multiple universities and regions in China. Such comparisons are especially important in the Chinese TNE context because cooperative programmes operate under broadly shared national regulations while differing substantially by region, institutional status, disciplinary configuration, and foreign partnership. China therefore provides a particularly valuable setting for examining how common policy frameworks interact with locally differentiated institutional conditions to shape student experience and satisfaction.

## 6. Conclusion

This study examined undergraduate student satisfaction in Sino-foreign cooperative programmes at a Chinese university with Australian, German, and US partners. It found significant differences by major, parental education, family income, and foreign partner institution, but not by gender, academic year, or place of origin. It also showed that student satisfaction was most closely associated with the teaching system, learning attitudes, and the quality of teacher-student and peer relationships.

The study contributes to research on TNE by showing that satisfaction in Chinese non-mobile programmes is best understood as a multidimensional and socially situated evaluation. Students did not evaluate their experience through international status alone. Instead, their judgments reflected the interaction between programme design, relational environment, and family resources. This reinforces the value of combining institutional, psychological, and sociological perspectives when analysing student experience in TNE.

The findings also carry practical implications. For universities, improving student satisfaction requires attention not only to external partnerships and programme branding, but also to the quality of everyday educational delivery. For researchers, the study highlights the need to take non-mobile pathways seriously as a central form of internationalisation in China. Overall, it suggests that student satisfaction in Chinese TNE appears less closely related to international labels themselves than to how international education is organised, experienced, and socially mediated within local institutional settings.

## Supporting information

S1 Dataset Minimal anonymised dataset underlying the quantitative findings reported in this study. (XLSX)
